# One-Day Interruption of NOAC Is Associated with Low Risk of Periprocedural Adverse Events during Pulmonary Vein Isolation If Combined with Left Atrial Thrombus Exclusion with Computed Tomography

**DOI:** 10.3390/life14010133

**Published:** 2024-01-17

**Authors:** Katalin Piros, Adorján Vida, Nándor Szegedi, Péter Perge, Zoltán Salló, Arnold Béla Ferencz, Vivien Klaudia Nagy, Szilvia Herczeg, Pál Ábrahám, Csaba Csobay-Novák, Zsófia Drobni, Tamás Tahin, Györgyi Apponyi, Béla Merkely, László Gellér, István Osztheimer

**Affiliations:** Heart and Vascular Center, Semmelweis University, Városmajor Street 68, 1122 Budapest, Hungary; piroskati90@gmail.com (K.P.); adorjan97@yahoo.com (A.V.); nandorszegedi@gmail.com (N.S.); peter.perge@gmail.com (P.P.); sallozoltan0121@gmail.com (Z.S.); ferenczarnold13@yahoo.com (A.B.F.); nagyklaudiavivien@gmail.com (V.K.N.); szilvi.herczeg@gmail.com (S.H.); palabrah@gmail.com (P.Á.); csaba@csobay.hu (C.C.-N.); zsofi.drobni@gmail.com (Z.D.); ttahin@gmail.com (T.T.); gyorgyi.apponyi@gmail.com (G.A.); merkely.bela@gmail.com (B.M.); laszlo.geller@gmail.com (L.G.)

**Keywords:** pulmonary vein isolation, NOAC interruption, patient comfort, computed tomography, transesophageal echocardiography

## Abstract

Background: Safety, efficacy, and patient comfort are the expectations during pulmonary vein isolation (PVI). We aimed to validate the combined advantages of pre- and periprocedural anticoagulation with non-vitamin K anticoagulants (NOACs) and rigorous left atrial appendage thrombus (LAAT) exclusion with computed tomography (CT). Methods: This study included a population of consecutive patients, between March 2018 and June 2020, who underwent cardiac CT within 24 h before PVI to guide the ablation and rule out LAAT. NOAC was omitted 24 h before the ablation. Results: A total of 187 patients (63% male) underwent CT before PVI. None of the patients experienced stroke during or after the procedure. The complication rate was low, with no thromboembolic events and 2.1% of patients experiencing a major bleeding event. Conclusions: Omitting NOAC 24 h before the ablation might be safe if combined with left atrial thrombus exclusion with computed tomography.

## 1. Introduction

It is known that atrial fibrillation (AF) increases the risk of stroke [1] and cognitive impairment [2]. In addition, AF is independently associated with all forms of dementia [3] and increased late mortality [4]. It also causes significant discomfort for the majority of patients. This includes palpitations, shortness of breath, and reduced physical performance. Catheter ablation (CA), instead of antiarrhythmic drug therapy, should be considered as the first-line treatment of symptomatic AF according to the current guidelines [5]. The cornerstone of AF ablation is pulmonary vein isolation (PVI) with continuous lesions around the veins in the antrum of the left atrium [6]. Continuous developments are ongoing to improve the safety and efficacy of the procedure [7,8,9].

The periprocedural management of oral anticoagulation is a crucial point during PVI. According to the current guideline, PVI is recommended to be performed with uninterrupted anticoagulation therapy [5]. However, it is acknowledged that in clinical practice, in the case of non-vitamin K oral anticoagulants (NOACs), one or two doses are usually omitted before ablation [10,11,12,13,14]. We acknowledge that the recommendations after our study do not recommend skipping NOAC the day before the intervention, so our study was not guideline-based [15]. However, they also emphasize the exclusion of a left atrial thrombus. A common feature of all PVI procedures is the delivery of a catheter into the left atrium from a transseptal approach. A catheter placed in the left atrium can mechanically mobilize any thrombus that may be there, causing a periprocedural stroke.

To warrant maximum safety and avoid periprocedural thromboembolic events, excluding left atrial thrombi during the preprocedural workup remains paramount. The gold standard for excluding a left atrial appendage thrombus (LAAT) is transesophageal echocardiography (TOE), a semi-invasive procedure. As shown by a meta-analysis, a reliable alternative method for LAAT exclusion is cardiac computed tomography (CT), which might be more comfortable for the patient and non-inferior to TOE [16]. In addition, CT images can be used before and during ablation to plan the procedure via image fusion with the intraprocedurally created electroanatomic map. Moreover, they might also provide additional information that can be useful in patient selection, for different PVI methods [17,18,19]. Some single-shot PVI procedures may be complicated by a short left common trunk. Some patients may have an accessory vein, which cannot be mapped reliably by electroanatomical navigation. In addition, coronary artery disease and extracardiac processes may also be detected during preoperative CT.

**Figure 1:**
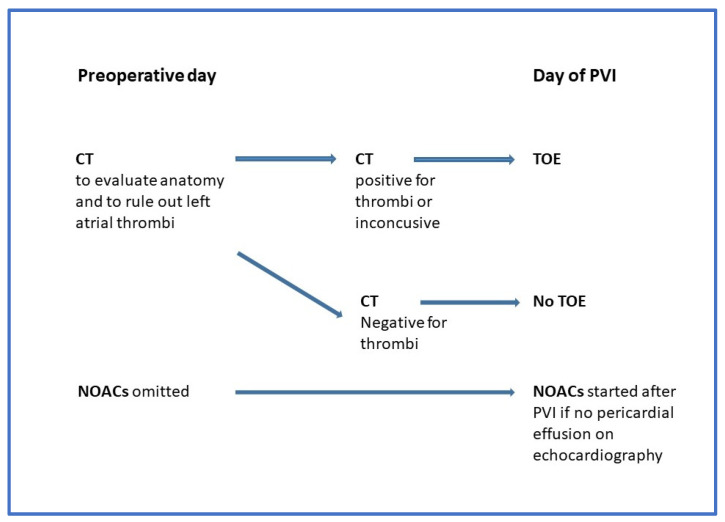
Workflow.

We aimed to validate the combined advantages of pre- and periprocedural anticoagulation with NOACs and rigorous LAAT exclusion with CT to keep periprocedural thromboembolic events at a minimal level and to lessen bleeding complications. We built the following workflow for patients undergoing PVI: CT was performed one day before or on the day of PVI for LAAT exclusion and specification of left atrial anatomy. All patients were anticoagulated with NOAC. NOAC was interrupted the day before and in the morning of PVI and continued on the day of the procedure after vascular complication, and pericardial effusion was ruled out with transthoracic echocardiography (Figure 1). If a bleeding complication was observed, the evening dose of NOAC was omitted, and Na-heparin was started after the complication resolved. We analyzed the periprocedural characteristics and the complication rate in patients undergoing PVI, having performed CT previously for LAAT exclusion. Our aim was not to detect statistical significance, as this would require a large randomized trial, which is difficult to perform according to the current guidelines. Rather, it is to support the utility of our clinical practice with clinical data. Such clinical data are rarely published, although they are of great use.

## 2. Materials and Methods

### 2.1. Patient Population

Our retrospective, single-center study included consecutive patients who underwent CT for LAAT exclusion 24 h before PVI between March 2018 and June 2020. Inclusion criteria were symptomatic patients with indication for PVI who were anticoagulated with NOACs and had not experienced any prior bleeding complication. Patients anticoagulated with vitamin K antagonists (VKAs) were excluded from the study.

Our center is a high-volume university teaching hospital. In 2018, 2019, and 2020, 626, 584, and 558 PVIs were performed in our institute. With this significant number of interventions, we were only able to include a small proportion of patients due to the above criteria. The main reason was the adjustment of the CT scan time to 24 h before the intervention. There is no CT shift on Sundays, and it was not possible to perform cardiac CT during the week every day. Also, some of the colleagues had the CT scan performed several days earlier to better plan the procedure. We decided to exclude patients with previous bleeding complications from the study because of the intention to create a homogeneous patient group.

Periprocedural characteristics and acute and late complications were assessed. Covariates of interest included patient demographics, medications, and standard cardiovascular risk factors (e.g., diabetes mellitus, hypertension, left ventricular function impairment, coronary artery disease, peripheral artery disease, previous stroke or transient ischemic attack, renal function, sex, age). Data relevant to atrial fibrillation, type of anticoagulation, bleeding complications, CHA_2_DS_2_-VASc score, and the specific anticoagulation treatments, including the use of NOACs or VKAs, were retrospectively collected by manual chart review. Data specific to the outcomes, bleeding, hospital stay, stroke/thromboembolic event, pericardial effusion, and tamponade were also collected retrospectively.

### 2.2. CT Examination

Coronary CTA was performed with a 256-slice multidetector CT (Brilliance iCT; Philips HealthTech, Best, The Netherlands). Prospective ECG-triggered non-contrast, contrast-enhanced, and delayed-phase scans were acquired.

For coronary artery calcium score, a 120-kV tube voltage with a 30–50 mAs tube current was used. For coronary CTA, a tube voltage of 100–120 kV with a 200–300 mAs tube current, depending on patient anthropometrics, was set. Image acquisition was performed with 128 mm × 0.625 mm detector collimation and 270 ms gantry rotation time.

Beta-blockers were administered to reach a target heart rate below 60/min. For heart rate control, 50–100 mg metoprolol was given orally and 5–20 mg metoprolol was given intravenously, if necessary. In patients with a heart rate of <80/min, mid-diastolic triggering was applied with 3–5% padding (73–83% of the R-R interval), and in those with ≥80/min, systolic triggering was chosen (35–45% of the R-R interval) regardless of the presence of AF in the ECG during CT examination. Sublingual nitroglycerin (0.8 mg) was administered on the table, a maximum of two minutes before the image acquisition. In total, 85–95 mL of contrast material (Iomeron 400, Bracco Ltd., Milan, Italy) was injected with a flow rate of 4.5–5.5 mL/s via the antecubital vein. A four-phasic contrast injection protocol was used. Bolus tracking in the LA was used to obtain proper scan timing. CT datasets were reconstructed with 0.8 mm slice thickness and 0.4 mm increments. Delayed-phase images were acquired 60–90 s after the contrast injection at the level of the left atrium with a smaller field of view to minimize the radiation dose.

All image analyses were performed offline on a dedicated workstation (Intellispace portal, Philips Healthcare), Best, The Netherlands. Non-contrast datasets were reconstructed with a slice thickness and increment of 2.5 mm, while coronary CTA datasets were reconstructed with a 0.8 mm slice thickness and 0.4 mm increment.

### 2.3. Ablation Procedure

In all patients who were on NOAC therapy started at least one month prior to ablation, NOAC was interrupted the day before and in the morning of PVI. If the patient was not anticoagulated before the PVI (CHA2DS2-VASc score 0), NOAC therapy was started on the evening of the procedure after the echocardiography.

All patients underwent standard-of-care pulmonary vein isolation procedures as follows: Conscious sedation was introduced using fentanyl and, if needed, with propofol. Patients underwent local anesthesia before the right femoral vein puncture was performed. Vascular ultrasound was not regularly used to guide femoral vein puncture. It was available in the lab and was used just in case a difficulty emerged during puncture. We recognize that regular ultrasound use is recommended based on the results of clinical trials. Our high-volume training center employs 6 qualified electrophysiologists and 5–7 fellows. Femoral puncture is the first step in manual training and is therefore regularly performed by our fellows. A trained electrophysiologist does not always supervise this step. The level of experience of the fellows varies widely. Electrophysiologists perform 60–150 PVIs per year.

Afterward, a decapolar catheter was placed into the coronary sinus. A double transseptal approach was used for radiofrequency (RF) ablation to introduce the mapping (Inquiry Optima, Abbott Plymouth, MN, USA, or Lasso Circular mapping catheter, Biosense Webster Irvine, CA, USA) and ablation catheter (TactiCath Contact Force Ablation Catheter, Abbott Plymouth, MN, USA or ThermoCool SmartTouch catheter, Biosense Webster Irvine, CA, USA) into the left atrium. The transseptal puncture was performed with SL0 (Abbott, Plymouth, MN, USA) sheaths and a Brockenbrough XS needle (Abbott) with fluoroscopy and pressure guidance. In some cases (n = 15), intracardiac echocardiography was used to aid safe transseptal puncture (left femoral vein access 10F introducer). If fluoro-guided transseptal puncture seemed to be difficult or some anatomy variants were seen on the CT image, ICE was used for the transseptal puncture. It was always an operator-dependent decision. Na-heparin was given immediately after the first transseptal puncture and titrated to a 300 s activated clotting time (ACT) level. The first transseptal puncture was changed over the wire to an Agilis (Abbott) steerable sheath, Point-by-point ablation was performed in the antral region of pulmonary veins. After ablation, entrance and exit blocks were confirmed during sinus rhythm. Entrance block was demonstrated with the aid of a lasso catheter placed in the ipsilateral vein, at which point the pulmonary vein potentials disappear. Exit block was demonstrated by stimulation from an ablation catheter placed in the same vein alongside a lasso catheter placed in the vein. Local capture (pulmonary vein capture) was demonstrated with exit block to the atria.

After successful PVI, the sheaths were removed immediately in the electrophysiology lab without the reversion of heparin action. A femoral pressure bandage was applied for 4 h. Echocardiography was performed immediately after the procedure in the electrophysiology lab, the day of the procedure before the first NOAC dose was given, and the day after the procedure before discharge home. We also performed echocardiography in case of complaints or hypotension. We actively monitored our patients for neurological symptoms after the procedure. We regularly assessed speech and limb movement.

We tested our patients for the presence of AV fistula. We examined the puncture site before release. If the examiner deemed it appropriate, he or she performed auscultation over the puncture site. However, this was not performed in the absence of complaints and visible complications. In all cases, a vascular ultrasound was performed if deemed necessary [20,21].

Fluoroscopy doses were measured and calculated using internationally used formulas: during ablation procedure: ED (mSv) = KAP (Gycm^2^) × 0.2 (mSv/Gycm^2^) [22]; during CTA: ED (mSv) = DLP (mGy × cm) × k, where k = 0.017 mSv/ mGy × cm [23].

### 2.4. Discomfort Study

Data about patient comfort and discomfort (discomfort study) during CT, PVI, and any previously performed TOE procedures (not necessarily during the PVI procedure, but a previous TOE-guided electrical cardioversion) were gathered retrospectively during telephone visits. The study is presented in the supplementary materials.

### 2.5. Postprocedural Anticoagulation

Echocardiography was performed after PVI to exclude pericardial effusion. The NOAC was restarted on the day of the PVI in the evening if no bleeding complication occurred. If a bleeding complication was observed and resolved, anticoagulation was restarted as soon as possible with heparin and titrated according to activated partial thromboplastin time (aPTI). NOAC was administered for at least three months post-ablation. Patients were discharged home the day after the procedure if there were no complications.

### 2.6. Follow-Up and Complications

All patients were scheduled for a follow-up visit three months after ablation.

Major complication was defined as periprocedural death and life-threatening or severe complications such as esophageal perforation/fistula, periprocedural thromboembolic event, cardiac tamponade, pulmonary vein stenosis, persistent phrenic nerve palsy, vascular complication, conversion to sternotomy, or pneumothorax. A vascular complication was considered as a major complication if it required intervention for treatment, caused long-term disability, or resulted in prolonged hospitalization. Other complications were considered minor.

- A periprocedural thromboembolic event was defined as any embolic event that occurred before or on the day of the ablation procedure or during the three-month period postprocedurally.
- Pericardial effusion was considered when an extra fluid buildup was observed in the pericardium after the ablation procedure with or without symptoms and did not cause heart failure or shock.
- Cardiac tamponade was defined as a compression of the heart causing heart failure and shock due to blood buildup in the pericardial space.
- Significant pulmonary vein stenosis was determined when a progressive lumen reduction of one or more pulmonary veins occurred, causing shortness of breath, cough, or hemoptysis.
- Persistent phrenic nerve palsy was diagnosed when fluoroscopy revealed paradoxical motion of the diaphragm.
- Inguinal hematoma was considered when a groin mass occurred after the removal of the sheets from the region, which was then observed and checked by ultrasound imaging to exclude more severe complications.
- Significant wound bleeding would be considered when the patient required extra hospital stay because of blood loss.
- Pseudoaneurysm was defined as a leakage of arterial blood into the surrounding tissue, with persistent communication visualized by ultrasound imaging.

### 2.7. Statistical Analysis

Continuous variables are presented as means and standard deviation (SD), while categorical parameters are shown as numbers and percentages. Continuous variables were compared using Student’s *t*-test, binary variables were compared using Fisher’s exact test, and categorical parameters were compared using the Chi-square test. In our cohort study, continuous variables showed normal distribution according to the Shapiro–Wilk normality test. A two-tailed *p*-value < 0.05 was considered significant. Statistical analysis was performed with GraphPad Prism, version 6.01 (GraphPad Software, Inc., La Jolla, CA, USA).

### 2.8. Informed Consent/Ethics Approval

All subjects gave their informed consent for inclusion before they participated in the study. The study was conducted in accordance with the Declaration of Helsinki, and the protocol was approved by the Semmelweis University Regional and Institutional Committee of Science and Research Ethics (No. 128/2020).

## 3. Results

One hundred eighty-seven patients (age 62 ± 11) were included in our study; 63% (n = 118) were male. The median CHA2DS2-VASc score was 2 (0–5). The baseline characteristics of patients are presented in Table 1. Notably, 11 patients (5.9%) had a previous stroke or TIA, and 17 (9.1%) were taking antiplatelet drugs. The antiplatelet drugs were given to patients with neurological and cardiological indications. No drug changes/optimization were performed during the study.

### 3.1. NOAC

Rivaroxaban was the most frequently used anticoagulant, accounting for 41.7% of the cases (Table 2). However, the use of all four available NOACs occurred among our patients.

### 3.2. CT

**Figure 2:**
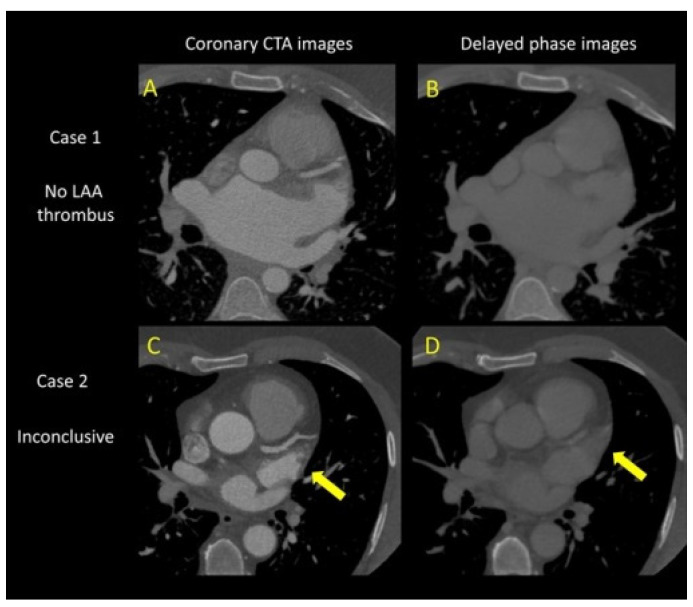
CTA image of the heart, with a yellow arrow indicating a possible left atrial appendage thrombus, which is more pronounced in the delayed-phase images. ((**A**) no thrombus Coronary CT, (**B**) no thrombus Delayed phase, (**C**): inconclusive for thrombus coronary CT, (**D**): inconclusive for thrombus Delayed phase).

In 41% (n = 76) of the cases, the CTA was performed on the day of PVI, while in the other cases, it was performed the day before PVI within 24 h. The CTA was not able to exclude the presence of LAAT (Figure 2) in 6% (n = 11) of the patients; thus, TOE was performed. The presence of LAAT was excluded in these cases with TOE. No LAAT was detected in the present population.

### 3.3. Ablation

The mean procedure time of PVI was 91.5 ± 24 min with 58.8 ± 19 min mean left atrial time. The mean fluoroscopy time was 330.5 ± 284 s with a 0.88 ± 0.94 mSv mean effective dose. The mean effective dose during CTA was 5.55 ± 2.8 mSv. ENSITE NavX and Carto 3 electroanatomical mapping systems were used in 56.7% (n = 106) and 43.3% (n = 81) of the cases, respectively.

### 3.4. Adverse Events

Minor acute complications were observed in nine cases (4.8%), and major acute complications were observed in four patients (2.1%); there were no acute or late ischemic complications during the three-month follow-up (Table 3). Inguinal hematoma was seen in five cases (2.7%), and pericardial effusion was seen in three cases (1.6%); these complications did not require further intervention. Significant bleeding from the femoral vein occurred in one case. Pericardiocentesis was needed due to tamponade in two cases (1.1%). In one case, pericarditis occurred, and it did not require further intervention. In one case, a thrombin injection was needed due to a femoral pseudoaneurysm.

## 4. Discussion

As already shown, CTA is a safe method for the exclusion of LAAT [24]. Although our study is underpowered for estimating the thromboembolic event rate with a one-day interruption of NOACs before PVI, we did not see any stroke event during the three-month follow-up of the patients. One-day interruption of NOAC before PVI together with rigorous thrombus exclusion might be a feasible approach.

Our study presents data from a real-world clinical practice in which NOAC is stopped one day before the procedure. It is very rare for such data to be published, as current guidelines do not recommend it.

In our study, the complication rate is similar to that published by high-volume centers, with no symptomatic thromboembolic events (see Table 4 for reference). Major bleeding events occurred in 1.6% (two tamponades and one femoral pseudoaneurysm) of the cases.

The best way to reduce the various bleeding complications is to avoid bleeding by using different imaging techniques. Ultrasound-guided puncture is the most suitable tool to avoid femoral complications [35]. Routine ICE use reduces the incidence of pericardial tamponade and other mechanical complications. In our study, neither vascular ultrasound nor ICE was routinely used, and femoral puncture and transseptal puncture were performed by a fellow in training. In light of this, our results show a very low complication rate.

Although we have not carried out an MRI study to investigate silent stroke, it is known that NOAC omission is associated with an increased rate of silent stroke. Likewise, procedure length and cardioversions also increase the rate of silent ischemia. At present, the impact of these findings on clinical neurological status remains to be clarified [36,37].

Our results are very similar to those reported previously in the international literature. It is important to highlight from our data that there was not a single thromboembolic complication. These complications are the most serious and the most difficult to treat, so their prevention is of paramount importance. Even in the NOAC era, active left atrial thrombus screening should not be omitted in our opinion. The use of CT for this purpose is safe. It can also help to better plan the intervention with knowledge of the pulmonary vein anatomy and to diagnose coronary and extracardiac abnormalities. Moreover, our data presented in the supplementary materials show that CT scans are not uncomfortable for our patients.

Our study has important limitations and is retrospective and single-center. In our opinion, our data may be valuable for improving clinical practices in the context of technical advances in atrial fibrillation ablation and the increasing use of newer and newer forms of anticoagulant therapy.

## 5. Conclusions

Based on our results, one-day interruption of NOAC before PVI might be safe if combined with rigorous left atrial thrombus exclusion with CT. Our data show no cases of thromboembolism despite the fact that patients did not receive NOAC therapy for one day prior to the PVI procedure.

## 6. Limitations

The main limitation of our study is the single-center and retrospective observational layout. Another limitation is the small sample size. For this reason, the clinical trial is clearly not strong enough.

## Figures and Tables

**Table 1:** Baseline characteristics of the patients. LVEF = left ventricular ejection fraction, GFR: glomerular filtration rate.

Baseline Characteristics (Mean ± SD)	Number of Patients (n = 187)
Age (years)	62 ± 11
Male sex	117 (62.6%)
LVEF < 30%	3 (1.6%)
Hypertension	133 (71.1%)
Coronary artery disease	35 (18.7%)
Diabetes	31 (16.6%)
Peripheral artery disease	4 (2.1%)
Prior transient ischemic attack/stroke	11 (5.9%)
GFR > 30	187 (100%)
Thrombocyte inhibitors	17 (9.1%)

**Table 2:** The distribution of different types of NOACs in the study population.

NOAC Type	Number of Patients (n = 187)
Rivaroxaban	78 (41.7%)
Apixaban	50 (26.7%)
Dabigatran	32 (17.1%)
Edoxaban	27 (14.4%)

**Table 3:** The type of complications and actions needed for resolution.

Type of Complication	Number of Patients (n = 187)	Action Needed
Inguinal hematoma	5 (2.7%)	no action needed
Wound bleeding	1 (0.5%)	one extra day observation
Pericardial effusion	3 (1.6%)	one extra day observation
Tamponade	2 (1.1%)	pericardiocentesis
Pseudoaneurysm	1 (0.5%)	percutaneous thrombin injection

**Table 4:** Comparison of our study results with other studies.

Study	Bridging	NOAC	Total Complications	Embolic Events	Vascular Complications
Current	No	All	6.9%	0%	3.8%
ESC EURObsrvational [25]	Yes (81%)	No	7.7%	0.6%	1.3%
Contemporary management of patients undergoing atrial fibrillation ablation [26]	Yes (63.8%)	23%	7.8%	0.3%	1.8%
Middelheim PVI- registry [27]	Yes	Yes	10.1%	0.1% *	4%
Repeat procedure is a new independent predictor of complications of atrial fibrillation [8]			2.82%	0.48% *	2.25%
Procedural success, safety and patients satisfaction after second ablation of atrial fibrillation [28]			12.6%	0.6% *	
CABANA [29]		32% †	8% †	0.3% *,†	3.8% †
Real-world comparison of in-hospital complications after catheter ablation for atrial fibrillation [30]		50%	2.3% ‡	0.5% ‡	0.2% ‡
RE-CIRCUIT [31]	No	≈50%	18.6% ‡	0% #	0.63% ‡
VENTURE-AF [32]	No	50%	20.6%	0% #	10.48% ‡
AXAFA-AFNET 5 [33]	No	≈50%	6.9% ‡	0.6% #	
ELIMINATE-AF [34]	No	66.9%	2.7% ‡	0.3% #	

* stroke, † catheter ablation subgroup, ‡ NOAC subgroup, # stroke on NOAC.

## Data Availability

Data are contained within the article.

## Supplementary Materials

The following supporting information can be downloaded at: https://www.mdpi.com/article/10.3390/life14010133/s1, Table S1: Distribution of inconvenient peri-ablation events according to QqL questionaries. TOE = transoesophageal echocardiography, CTA = computed tomography angiography. Figure S1: Discomfort score comparing TOE, ablation procedure and CT. TOE = transoesophageal echocardiography, CT = computed tomography.
