# Supplementary material for: One-Day Interruption of NOAC Is Associated with Low Risk of Periprocedural Adverse Events during Pulmonary Vein Isolation If Combined with Left Atrial Thrombus Exclusion with Computed Tomography
Source: Life (Basel). 2024 Jan 17;14(1):133. doi: 10.3390/life14010133 (PMC10817453; doi:10.3390/life14010133)
Supplement: Supplementary file 1 [file life-14-00133-s001.zip › life-2770777-supplementary.pdf]

Supplementary Material:

Materials and Methods: Evaluation of procedural discomfort:

Data about patient comfort and discomfort (Discomfort study) during CT, PVI, and any previously performed TOE procedures (not necessarily during the PVI procedure, but a previous TOE guided electrical cardioversion) were gathered retrospectively during telephone visits. We did not want to collect data during the procedure, but rather interviewed our patients after the whole procedure. Patients were asked to choose which of the following was the worst: atrial fibrillation, the TOE, hospital stay, ablation procedure, the CT. Also, we asked to estimate the pain/inconvenience caused by the ablation procedure, by the TOE, and by CT on a scale of 1 to 10.

Results: Discomfort:

From our cohort, 67% of the patients could be contacted for the Discomfort study. According to the patient's Discomfort study, the discomfort caused by the atrial fibrillation itself was the worst (70, 55.6%), followed by the TOE, then the ablation procedure hospital stay, while the CT examination was the least inconvenient for the patients (Table S1). Results comparing TOE, ablation procedure, and CTA with numerical answers showed the following: TOE received the highest inconvenience scores (5.08), followed by the ablation procedure (3.49), and the least inconvenient proved to be the CTA (2.32). Statistical significance was present comparing CTA vs. TOE ($p<0.0001$), CTA vs. ablation procedure ($p<0.0001$), and ablation procedure vs. TOE ($p=0.006$). (Figure S1)

Table S1. Distribution of inconvenient peri-ablation events according to QqL questionaries. TOE= transoesophageal echocardiography, CTA= computed tomography angiography.

Event	Number (percentage) of patients considering the given event the worst (n=126)
atrial fibrillation	70 (56%)
TOE	22 (17%)
ablation procedure	17 (13 %)
hospital stay	12 (10%)
CTA	5 (4%)

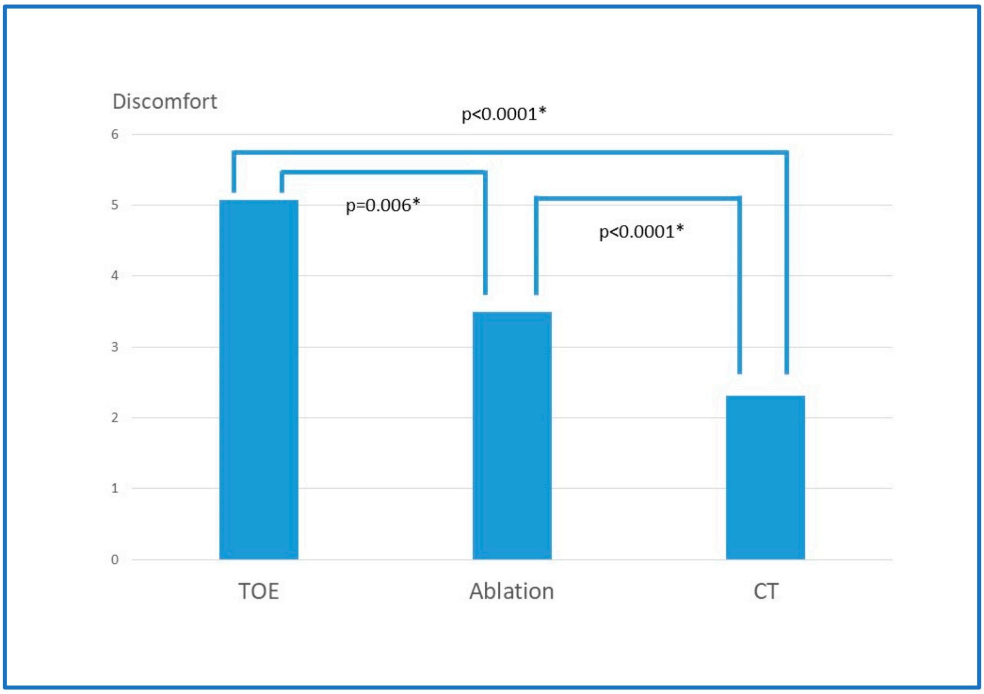

Figure S1. Discomfort score comparing TOE, ablation procedure and CT. TOE= transoesophageal echocardiography, CT= computed tomography.

Discussion

It is a good testament to our patient selection that the most common complaint for our patients is atrial fibrillation itself. It is in this patient population that ablation has the greatest clinical benefit. The discomfort of arrhythmia also steers our patients towards ablation. It is very important not to cause more discomfort to our patients with ablation and perioperative interventions than the arrhythmia itself. Although much less unpleasant, the TOE examination is the worst sensation after the arrhythmia.

Conclusion:

The inconvenience caused by periprocedural examinations can be significantly reduced by replacing the TOE examination with CT, which is suitable to exclude LAA thrombus in most cases. This is also very important as a significant proportion of patients may undergo multiple ablations. This requires gaining the patient's trust and consent for a second ablation. If the procedure can be performed without pain and discomfort, we can win the trust of our patients for the long term. However, atrial fibrillation itself is the biggest complaint, with all other medical interventions and diagnostic procedures being less unpleasant.
